# Non-Invasive Analysis of Recombinant mRNA Stability in *Escherichia coli* by a Combination of Transcriptional Inducer Wash-Out and qRT-PCR

**DOI:** 10.1371/journal.pone.0066429

**Published:** 2013-06-19

**Authors:** Veronika Kucharova, Trine Aakvik Strand, Eivind Almaas, Adrian E. Naas, Trygve Brautaset, Svein Valla

**Affiliations:** 1 Department of Biotechnology, Norwegian University of Science and Technology, Trondheim, Norway; 2 Department of Molecular Biology, SINTEF Materials and Chemistry, Trondheim, Norway; Florida International University, United States of America

## Abstract

mRNA stability is one among many parameters that can potentially affect the level of recombinant gene expression in bacteria. Blocking of the entire prokaryotic transcription machinery by addition of rifampicin is commonly used in protocols for analysis of mRNA stability. Here we show that such treatment can be effectively replaced by a simple, non-invasive method based on removal of the relevant transcriptional inducers and that the mRNA decay can then be followed by qRT-PCR. To establish the methodology we first used the *m*-toluate-inducible XylS*/Pm* expression cassette as a model system and analyzed several examples of DNA modifications causing gene expression stimulation in *Escherichia coli*. The new method allowed us to clearly discriminate whether an improvement in mRNA stability contributes to observed increases in transcript amounts for each individual case. To support the experimental data a simple mathematical fitting model was developed to calculate relative decay rates. We extended the relevance of the method by demonstrating its application also for an IPTG-inducible expression cassette (LacI/*P_tac_)* and by analyzing features of the bacteriophage T7-based expression system. The results suggest that the methodology is useful in elucidating factors controlling mRNA stability as well as other specific features of inducible expression systems. Moreover, as expression systems based on diffusible inducers are almost universally available, the concept can be most likely used to measure mRNA decay for any gene in any cell type that is heavily used in molecular biology research.

## Introduction

The use of inducible promoter systems for heterologous protein expression in *Escherichia coli* is one of the most convenient and actively used methods, accounting for one third of protein production altogether [1]–[3]. The inducible *Pm* promoter regulates the expression of the *meta*-cleavage pathway operon involved in the catabolism of aromatic hydrocarbons in the TOL plasmid (pWWO) of *Pseudomonas putida* [4]. Together with its positive regulator XylS that is activated by the binding of downstream products in the pathway (alkylbenzoates), the *Pm* promoter system has been established in our laboratory as a robust model for heterologous expression in various bacterial hosts [5]–[7]. An important factor controlling the quantity of heterologous protein being produced is the cellular concentration of its mRNA, shaped by the synthesis and degradation rates [8]. The efficiency of transcription has often been one of the first intuitive steps to target in order to stimulate recombinant gene expression [9]–[11], and for the XylS*/Pm* expression system this has been accomplished via directed evolution of the *Pm* promoter region [12] and the coding sequence of the XylS transcriptional regulator [13]. In addition, strong stimulation of the transcript levels has also been achieved by mutating the *Pm* promoter associated 5′ untranslated region (UTR) or by fusing translocation signal sequence to a heterologous gene [14], [15].

The mRNA degradation processes in bacteria are complex and not yet fully understood. The originally proposed mRNA degradation model correctly suggested that the mRNA lifetimes in bacteria are controlled primarily by internal endonuclelyotic cleavage followed by 3′–exonuclease attack [16], [17], but failed to explain the influence of 5′-terminal ends on mRNA half-lives [18]–[20]. Two recent studies re-examined the initial events of RNA decay and uncovered that mRNA degradation can be triggered by pyrophosphate removal at the 5′-terminal end [21], [22], a modification which makes it a preferred substrate for the essential RNase E [23]. The importance of understanding the factors that affect mRNA stability for applications in biotechnology and metabolic engineering is widely recognized [24]–[27], and established methods for assessing the stability of mRNAs in prokaryotes commonly involve the use of antibiotic inhibitors of transcription (particularly rifampicin) combined with pulse-chase procedures, Northern-blot analyses and microarrays. The use of rifampicin turns off most or all transcription initiation in the cells, which can lead to serious effects on bacterial cells [28], making it virtually impossible to exclude unintended (and possibly also mRNA-specific) effects on transcript decay. Our goal in this work was therefore to develop an alternative, non-invasive procedure that could be used for monitoring mRNA decay in a more controlled manner.

We have previously introduced a quantitative real time polymerase chain reaction (qRT-PCR) based method to study the kinetics of recombinant transcript accumulation from the XylS/*Pm* expression system in *E. coli*, from the point of induction until the transcript reaches its steady-state level [14]. Here we describe how a methodologically related approach can be used to determine the decay kinetics of recombinant transcripts, allowing for evaluation of the mRNA stability. The procedure first involves a rapid wash-out of the transcriptional inducer, which results in a controlled and specific shut off of transcription of the recombinant gene. With the recombinant transcript synthesis stopped, its decay can be followed as a function of time by qRT-PCR. This second part can also be used in a rifampicin-based protocol. The assay is applicable for *E. coli* expression systems induced by small molecules able to diffuse in or out of the cells, such as benzoic acid and derivatives thereof that are used for induction of the XylS/*Pm* and XylR/*Pu* expression systems [7], [29], [30]. The assay should also be applicablefor the widely used IPTG inducer applied for example for induction of the LacI/*P_tac_* expression cassette. The protocol described here should not be restricted to the selected model host (*E. coli*), but most likely have a broad application potential in studies of mRNA decay in many types of organisms.

## Materials and Methods

### Growth Conditions and DNA Manipulations

*E. coli* strains were generally grown at 37°C in Luria-Bertani (LB) broth (10 g/L tryptone, 5 g/L yeast extract, and 5 g/L NaCl) or on LB agar (LB medium with 15 g/L agar) supplemented with ampicillin (200 mg/L) or kanamycin (50 mg/L) when appropriate. For expression experiments, recombinant cells were grown at 30°C and induction of the XylS*/Pm* or T7 and LacI/*P_tac_* system was done by adding *m*-toluic acid or IPTG, respectively, to the final concentrations as indicated in the text. When rifampicin was used for inhibiting transcription, it was added to a final concentration of 100 mg/L (90 minutes after induction at OD_600_ = 0.5) using a rifampicin stock in 95% ethanol at 10 g/L. Standard recombinant DNA procedures were performed as previously described [31]. Custom PCR primers were supplied by Eurofins MWG operon or Sigma-Aldrich Co. Spiked oligonucleotide mixtures used for combinatorial library constructions were supplied by Medprobe AS. DNA sequencing was performed by Eurofins MWG operon.

### Biological Materials

The strains and plasmids used in this study are listed in **Table 1** and the primers are listed in **Table 2**. Vectors pBS_2_P1bla and pBSP1bla-C19 were made from pBSP1bla, by replacing the NdeI-NcoI fragment containing the *bla* 5′ coding region, with annealed oligonucleotides corresponding either to the original sequence, except for a second codon change from AGT to TCT, or to a synonymous variant C19. Vectors for expression of recombinant proteins under control of the *P_tac_* promoter were based on the commercial pFLAG-CTC™ expression vector. The *gm-csf-c-myc-his_6_* and *ompA-gm-csf-c-myc-his_6_* coding regions were PCR-amplified from plasmid pGM29 or pGM29ompA, respectively, by using primer pair PmUTR.fwd/GOI.rev. The resulting DNA fragments were NdeI/KpnI digested, and ligated into corresponding sites of the multiple cloning site of pFLAG-CTC™, yielding pFLAG-OGM and pFLAG-GM vectors.

**Table 1:** Bacterial strains and plasmids.

Strain or plasmid	Description^1^	Reference
*E. coli strain*		
DH5α	General cloning host	Bethesda Research Laboratories
NEB Express Iq	Strain with enhanced expression of LacI repressor	New England Biolabs
ER2566	Strain with intracellular production of T7 DNA polymerase	New England Biolabs
*Plasmid*		
pIB11	RK2-based expression vector containing the XylS*/Pm* expression cassette, wild type *Pm*UTR and *bla* as the reporter gene for *Pm*; Km^r^; 8.1 kb	[12]
pIB11 LV-2	pIB11 derivative containing mutated 5′-UTR DNA sequence LV2 upstream of the*bla* gene; Km^r^; 8.1 kb	[14]
pIB11 LII-11	pIB11 derivative containing mutated 5′-UTR DNA sequence LII-11 upstream of the *bla* gene; Km^r^; 8.1 kb	[14]
pGM29	RK2-based vector expressing GM-CSF-c-myc-his_6_ fusion protein from theXylS*/Pm* expression cassette; Ap^r^; 8.7 kb	[15]
pGM29ompA	RK2-based vector expressing ompA-GM-CSF-c-myc-his_6_ fusion protein from theXylS*/Pm* expression cassette; Ap^r^; 8.8 kb	[15]
pIFN30S	RK2-based vector expressing IFN-α2b-c-myc-his_6_ fusion protein from the XylS*/Pm* expression cassette; Ap^r^; 8.8 kb	[15]
pIFN30SpelB	RK2-based vector expressing pelB-IFN-α2b-c-myc-his_6_ fusion protein from theXylS*/Pm* expression cassette; Ap^r^; 8.9 kb	[15]
pBSP1bla	RK2-based expression vector containing the XylS*/Pm* expression cassetteand *bla* as a reporter gene for *Pm*; Km^r^; 9.5 kb	[58]
pBS_2_P1bla	pBSP1bla derivative containing synonymous mutation in the 2^nd^ codon ofthe *bla* gene; Km^r^; 9.5 kb	This study
pBSP1bla-C19	pBSP1bla derivative containing synonymous *bla* variant C19; Km^r^; 9.5 kb	This study
pFLAG-CTC™	Cloning vector (f1 replicon) for expression of recombinant proteins fromthe LacI/*P_tac_* cassette. Ap^r^; 5.4 kb	Sigma-Aldrich Co.
pFLAG-GM	pFLAG-CTC™ derivative expressing GM-CSF-c-myc-his_6_ fusion protein; Ap^r^; 5.9 kb	This study
pFLAG-OGM	pFLAG-CTC™ derivative expressing ompA-GM-CSF-c-myc-his_6_ fusion protein; Ap^r^; 5.9 kb	This study
pSB-E2r	pMB1-based vector expressing IL1-RA protein from the LacI/*P_T7lac_* expression cassette; Km^r^; 6.1 kb	[59]
pSB-P2r	pMB1-based vector expressing IL1-RA protein from the XylS*/Pm* expression cassette; Km^r^; 5.8 kb	[59]

1 Ap^r^: ampicillin resistance gene; Km^r^: kanamycin resistance gene.

**Table 2:** PCR and qRT PCR primers.

Name	Sequence	Gene^1^
PmUTR.fwd	5′-AAGAAGCGGATACAGGAGTG-3′	*gm-csf/ompA-gm-csf*
GOI.rev	5′-CTTGGTACCTTGTTCGGCCGGAT-3′	*gm-csf/ompA-gm-csf*
bla.fwd	5′-ACGTTTTCCAATGATGAGCACTT-3′	*bla*
bla.rev	5′-TGCCCGGCGTCAACAC-3′	*bla*
gm-csf37.fwd	5′-CCCTGGGAGCATGTGAATG-3′	*gm-csf/ompA-gm-csf*
gm-csf111.rev	5′-CATCTCAGCAGCAGTGTCTCTACTC-3′	*gm-csf/ompA-gm-csf*
gm-csf226.fwd	5′-GGCCCCTTGACCATGATG-3′	*gm-csf/ompA-gm-csf*
gm-csf301.rev	5′-TCTGGGTTGCACAGGAAGTTT-3′	*gm-csf/ompA-gm-csf*
ifn-*α*2b324.fwd	5′-CGAGACCCCGCTGATGAA-3′	*ifn-α2b_S_/pelB-ifn-α2b*
ifn-*α*2b396.rev	5′-CAGATACAGGGTGATACGCTGAAA-3′	*ifn-α2b_S_/pelB-ifn-α2b*
km.fwd	5′-TACCTTTGCCATGTTTCAGAAACA-3′	Km^r^
km.rev	5′-AATCAGGTGCGACAATCTATCGA-3′	Km^r^
il-1-ra.rev	5′-TCAGACACATTTTACCACCAT-3′	*IL1RN_S_*
il-1-ra.fwd	5′-ATTGATGTGGTGCCGATTGA-3′	*IL1RN_S_*
16sRNA.fwd	5′-ATTGACGTTACCCGCAGAAGAA-3′	16 sRNA
16sRNA.rev	5′-GCTTGCACCCTCCGTATTACC-3′	16 sRNA

1 Km^r^: kanamycin resistance gene.

### Construction of Combinatorial Synonymous Libraries in the 5′ End of the *bla* Coding Sequence

A synonymous codon library of the *bla* 5′ coding sequence (SI library) was constructed in the pBSP1bla plasmid by replacing the sequence corresponding to the first 23 codons by synthetic oligonucleotide mixtures, followed by screening on solid media according to previously described methods [12], [14]. 12 different nucleotide mixtures, shown in the nucleotide sequences by the numbers 1–12, were used to synthesize doped oligonucleotide mixures: 1∶79% A +7% C +7% G +7% T, 2∶7% A +79% C +7% G +7% T, 3∶7% A +7% C +79% G +7% T, 4∶7% A +7% C +7% G +79% T, 5∶80% A +20% G, 6∶80% C +20% T, 7∶20% A +80% G, 8∶20% C +80% T, 9∶80% A +20% T, 10∶20% A +80% C, 11∶20% C +80% G, and 12∶10%A +10% C +80% T. The SI library was generated from the oligonucleotide mixture defined by 5′-TATG9(11)4AT(12)CA5CA8TT6(10)G4GT2GC26T4AT(12)CC2TT8TT8GC3GC1TT8TG66T4CC4GT4TT8GC-3′ and the non-coding strand corresponding to the original sequence. Because a synonymous change of the *bla* second codon (AGT → TCT) was particularly effective for increasing ampicillin tolerance level, a second library (SII library) was generated in the pBS_2_P1bla vector, using the synonymous second codon as the new start sequence. SII was constructed from the oligonucleotide mixture defined by: 5′- TATGTC4AT(12)CA5CA8TT6CG4GT2GC2CT4AT(12)CC2TT8TT8GC3GC1TT8TG6CT4CC4GT4TT6GC-3′. The non-coding strand was kept complementary to the original sequence, except for the TCT change in the second codon. Approximately 400 000 transformants containing synonymous *bla* mutants were mixed to constitute the SI library, while the SII library contained approximately 140 000 transformants. Screening for mutated *bla* 5′ coding sequences that result in increased ampicillin-tolerance levels was performed according to a previously described procedure [14].

### Determination of the Kinetics of Recombinant Transcript Accumulation

LB medium was inoculated to final OD_600_ = 0.05 with an overnight culture and grown at 30°C (220 rpm) until mid-log phase. At OD_600_ = 0.5 either 0.5 mM *m*-toluate (XylS*/Pm*) or 0.5 mM IPTG (LacIq*/P_tac_* and T7) was added immediately after the first sample (time zero) was collected. Samples for total RNA isolation collected at time points 0, 3, 5, 8, 10, 15, 20 and 60 minutes were processed as described in the qRT-PCR analysis chapter. For immediate stabilization, samples were treated with RNAprotect cell reagent (QIAGEN) prior to freezing.

### The Inducer Wash-out Method

20 mL of recombinant cultures were grown and induced as described above. Culture growth was continued for another 90 minutes, allowing the investigated mRNA to reach its steady-state level. 5 mL of the cultures were subsequently concentrated by rapid filtration through a Millipore EZ-Pak® Membrane filter (Millipore) with the use of a vacuum pump. The filter with harvested cells was washed with 10 ml of phosphate buffered saline (137 mmol/L NaCl, 2.7 mmol/L KCl, 8.1 mmol/L Na_2_HPO_4_• 2H_2_O, 1.76 mmol/L KH_2_PO_4_, pH 7.4), followed by resuspension of the cells in 10 mL of fresh 30°C pre-warmed LB medium without inducer and maintaining the culture growth for another 10 to 30 minutes. Samples for qRT-PCR analysis were taken directly after filter transfer and cell resuspension (zero minute sample) and at several time points after the filter transfer. Each sample was immediately after collection treated with the stabilizing RNAprotect cell reagent (QIAGEN).

### qRT-PCR Analysis

The analysis of transcript levels from different recombinant genes was performed as described previously [31]. A fragment from the 16S rRNA gene was used as a normalizer. All experiments were repeated at least twice, and measurements were carried out with minimum three technical recurrences.

### High-performance Liquid Chromatography (HPLC) Analysis of *m*-toluate

To determine the presence of *m*-toluate in cell culture samples HPLC analysis was performed as previously described [32], except that the temperature of the Aminex HPX-87-H column was changed to 55°C. Standards of *m*-toluate in LB media (0.5 and 0.2 mM) were used for calibration. For detection of *m*-toluate in the cells, recombinant cultures were harvested by centrifugation (3 ml), and cell free extracts were obtained by suspending the cell pellets in 0.9% NaCl and using a Branson Sonifier DSM tip for cell disruption (sonication for 3 minutes on ice, duty cycle 30% and output control 2.5). Samples were filtered through 0.2 µm Filtropur S syringe filters (Sarstedt) prior to HPLC analysis.

### Gas Chromatography-Mass Spectrometry (GC-MS) Analysis of Isopropyl β-D-1-thiogalactopyranoside (IPTG)

Agilent 7890A GC coupled to Agilent 5975 inert MSD was used for IPTG GC-MS analysis. Aqueous IPTG samples/standards (50 µL) were added to 25 µL stock solution of 11.75 mM Myristic-d-27 acid in solvent mixture water:methanol:isopropanol (2∶5∶2 (v/v/v)). Myristic acid served as internal standard. Samples/standards were vacuum dried using a Thermo Speed-Vac evaporator at 65°C, followed by derivatization according to Agilent Fiehn GC/MS metabolomics RTL library user guide (Agilent Technologies). Analysis was performed according to the guide with a modified temperature program: 60°C for 1 min, then 20°C/min to 325°C, final temperature held for additional 5 min.

### β-lactamase Enzymatic Assay and Quantification of Recombinant Proteins by SDS-PAGE/Western Blot Analysis (β-lactamase and GM-CSF)

For analysis of recombinant protein production level, an overnight culture was diluted with LB media to OD_600_ = 0.05 and grown at 30°C (220 rpm). Exponentially growing cells (OD_600_ = 0.1) were then induced either with *m*-toluic acid or IPTG (0.5 mM). Cell growth was continued for 5 hours after which recombinant cultures were harvested by centrifugation (6000 rpm, 5 minutes, 4°C, Eppendorf Centrifuge 5810R). Crude extracts were prepared by sonication 4×90 seconds, with 30 seconds cooling periods (Branson sonifier, 30% duty control, 3 output control). Total protein concentration was determined with the Bio-Rad Detergent-Compatible Protein Assay (Bio-Rad Laboratories, Hercules, CA, USA) as described by the manufactures. β-lactamase assay was performed according to the procedures previously described [33]. All enzyme activity analyses were repeated at least twice, and measurements were carried out with minimum three technical recurrences. Qualitative detection of β-lactamase and GM-CSF was performed by using SDS-PAGE and Western blot essentially as previously described [34], [35] except that direct detection with HisProbe™-HRP (Thermo Scientific) was applied for the His-tagged GM-CSF protein. Signals were developed by using Pierce ECL Western blot substrate for chemiluminescent detection according the manufacturer’s instructions. Chemiluminescence was detected by ChemiDoc™ XRS Imaging System and analyzed by Image Lab 4.0 software (Bio-Rad Laboratories.).

### Computational Estimation of mRNA Synthesis and Degradation Rate

Similar to the approach in [14], we assume that recombinant transcripts 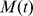 are synthesized after induction at a constant rate 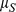 and degraded at a constant rate 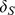:
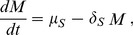
(1)with the general solution
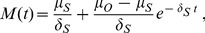
(2)where 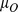 is the synthesis rate of the non-induced promoter. Assuming constant synthesis and degradation rates after transcriptional shut off, transcript decay is given by:
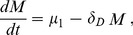
(3)with general solution
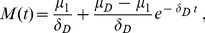
(4)where the rate 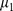 corresponds to leakage transcription in the inhibited promoter. We estimated the synthesis rate 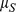 and the decay rate 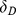 by employing non-linear least squares fitting using the Levenberg-Marquardt algorithm [36] on Eqn. (2) and Eqn. (4) respectively. All reported rate values are in units of “per minute”. The robustness of our parameter estimates was analyzed by calculating their 95% confidence intervals (95% CI) and conducting Jackknife sampling.

## Results

### The Use of UTR Mutants as Models to Study *bla* mRNA Stability by the Inducer Wash-out Method

Chemical inducers of recombinant gene expression are typically low molecular weight compounds that may or may not diffuse in and out of the cells. The inducer of the XylS/*Pm* system (*m*-toluate) enters *E. coli* independently of the cells transport systems and was therefore used as a first test of the wash-out concept. A series of initial experiments showed that a simple filtration procedure can be used to rapidly replace the growth medium of an induced cell culture by fresh medium lacking inducer. We then further confirmed the robustness of the method at different levels, as follows (data not shown): 1) by HPLC it was shown that the inducer is removed from both the media and cells after the filtration; 2) two independent primer pairs targeting the 5′ or the 3′-terminal end, respectively, were found to give corresponding results during qRT-PCR quantifications of the same recombinant mRNA; 3) it was demonstrated that the amounts of an mRNA produced constitutively (plasmid-borne kanamycin resistance gene) were not affected by the wash-out procedure itself.

**Figure 1:**
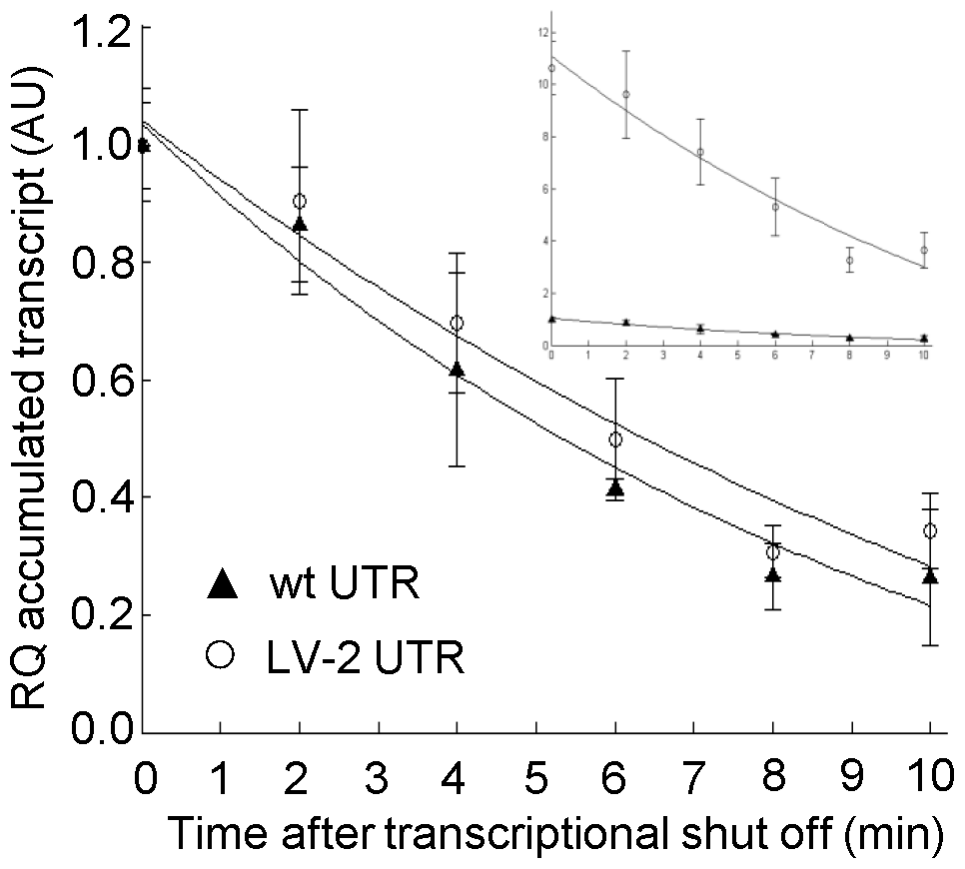
Decay kinetics of two *bla* transcripts with different 5′ UTR sequences. The respective genes were expressed from *Pm* and contained the DNA sequence of either the wild type 5′-UTR or the LV-2 variant. The larger plot describes decay kinetics where the level of the two *bla* transcripts is arbitrary set to one at time point zero. In the upper right corner the transcript amounts are represented with all values relative to the wild type 5′-UTR; arbitrary set to one at time point zero. Solid lines represent the best fit to the data calculated according to Eqn 4 (Material and Methods). Error bars show the deviation between three biological recurrences. RQ: relative quantification, AU: arbitrary units.

To confirm that the wash-out method could be used to analyze mRNA stability we first reinvestigated an earlier study showing strong stimulation of transcript accumulation resulting from specific mutations (LV-2 variant) in the DNA region corresponding to the 5′-UTR mRNA sequence of the *bla* reporter gene, encoding β-lactamase [14]. In this report it was concluded that the mutations were primarily acting by stimulating the transcription process itself, and not by increasing the mRNA stability. However, these conclusions did not follow from direct measurements of decay rates, and here we wanted to use this case for an initial evaluation of the inducer wash-out method. The *bla* gene was again used as the reporter (*E. coli* strains DH5α (pIB11) and DH5α (pIB11 LV-2)), and qRT-PCR quantifications of the mRNA levels at consecutive time points after inducer removal revealed that the decay kinetics is indeed similar for both transcripts (wild type UTR and LV-2 variant), despite a more than 10-fold difference in the accumulated transcript amounts (Figure 1). Subsequent mathematical fitting of the experimental data estimated the relative decay rates (defined by Eq. 4 in Material and Methods) to be 0.14 for the wild type (95% CI = 0.13–0.17) and 0.13 for the LV-2 variant (95% CI = 0.12–0.16). These results were consistent with the previous findings, and therefore encouraged us to study more cases, involving less predictable outcomes.

In the study in which the LV-2 mutant was identified we also isolated a number of other UTR variants which appeared to stimulate translation more selectively, and we selected such a variant (LII-11) as an example for the next mRNA stability examination. Generally it is known that efficient translation may lead to protection against mRNA degradation, presumably due to a higher ribosomal occupancy of the transcript and steric protection of potential RNase E cleavage sites [37], [38]. Interestingly, the kinetics of the LII-11 transcript degradation could not be distinguished from that of the wild type UTR or the LV-2 variant (data not shown). Thus, this result suggested that stimulated translational efficiency does not necessarily lead to an increased mRNA stability. Even though the LII-11 UTR variant led to a stronger stimulation at the protein than at the mRNA level (in contrast to LV-2), more transcripts (compared to the wild type) also accumulated when *bla* was expressed with the LII-11 UTR [14]. The stability analysis reported here may therefore be interpreted to mean that LII-11 lead to a mixture of transcriptional and translational stimulation and that the translational stimulation is not accompanied by a corresponding protection of the transcript from degradation.

### Certain Synonymous Codon Changes at the 5′ End of the *bla* Coding Sequence Stimulate the Gene Transcription without Affecting mRNA Stability

**Figure 2:**
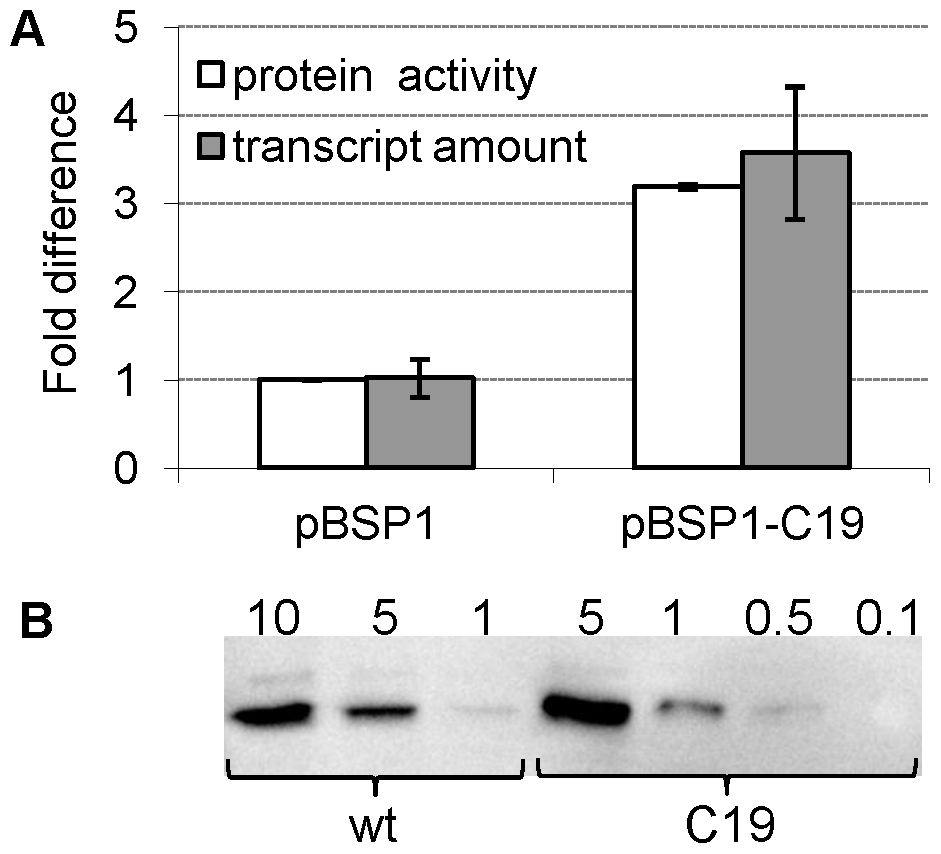
The *bla* gene expression in DH5α (pBSP1bla) and DH5α (pBSP1bla-C19). **A.** Determination of accumulated transcript levels and enzymatic activity of β-lactamase, with all values relative to the pBSP1bla level (arbitrarily set to one). **B.** Total β-lactamase production of DH5α (pBSP1bla) (denoted as wt) and DH5α (pBSP1bla-C19) (denoted as C19) was visualized by Western blotting and total cell samples were loaded in dilution series of decreasing total protein amount (µg). The size of the mature β-lactamase protein is approximately 29 kDa.

**Figure 3:**
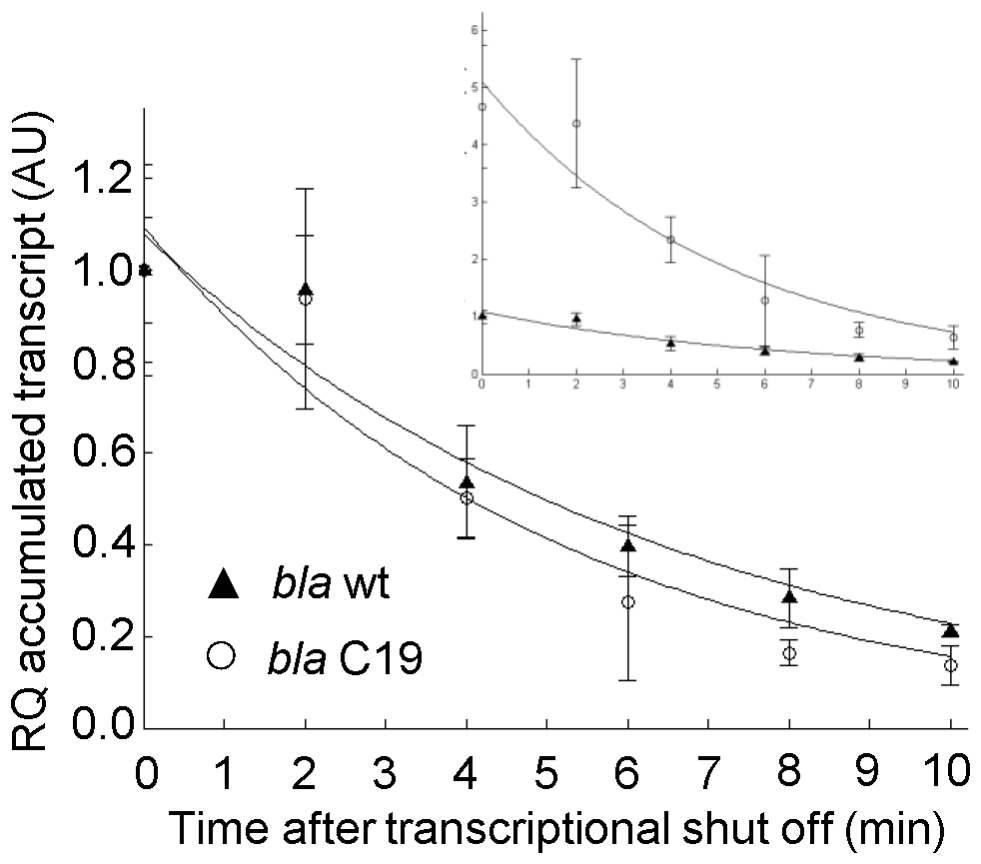
Kinetics of transcript decay for two variants of the *bla* gene. Synonymous codon variant C19 compared to the *bla* wild type, expressed from *Pm*. In the larger plot transcript amounts at time point zero for the wild type *bla* mRNA and the C19 variant are arbitrarily set to one. In the upper right corner the transcript amounts are shown with all values relative to the wild type *bla* transcript amount at time point zero (arbitrarily set to one). Solid lines represent the best fit to the data calculated according to Eqn 4 (Material and Methods). Error bars show the deviation between three biological recurrences. RQ: relative quantification, AU: arbitrary units.

Since complete genes can now be synthesized commercially at a reasonable cost, optimization of codon usage has become a popular strategy to enhance expression of poorly expressed genes [39]–[41]. The total number of possible synonymous codon variants for a complete gene is enormous, but here we decided to specifically study the effects of codon changes limited to the 5′ end of the *bla* coding sequence. The advantage of using this particular gene for such analyses is that mutants with stimulated β-lactamase production can be easily selected by the ability of the corresponding host cells to grow on agar medium supplied with elevated levels of ampicillin [12], [14]. We constructed and screened a library of such mutants (Materials and Methods) and one of them displayed an approximately 9-fold increase in the host ampicillin tolerance level. This mutant was designated C19 and contained a 2^nd^ codon change from AGT to TCT (Serine) and a 8^th^ codon change from GTC to GTT (Valine). Analysis of *bla* transcript amounts and β-lactamase activity (strains DH5α (pBSP1bla) and DH5α (pBSP1bla-C19)) indicated more than 3-fold increase for both, relative to the wild type (Figure 2A). Western blot analysis showed that the increase in enzyme activity is based on increased protein production, as a good correlation between the enzyme amount and corresponding activity level was observed (Figure 2B). The observed increase in accumulated transcripts might potentially be the result of enhanced mRNA stability, but the corresponding *bla* mRNA decay kinetics showed that this is not the case (Figure 3; relative decay rates: 0.16 for the wild type (95% CI = 0.14–0.18) and 0.19 for the C19 variant (95% CI = 0.15–0.23)). Surprisingly, these results therefore indicated that the mutations in the coding sequence can result in stimulation of transcription, without significantly affecting mRNA stability.

### Fusion of a 5′ Translocation Signal Sequence to a Heterologous Gene can Confer Increased mRNA Stability

**Figure 4:**
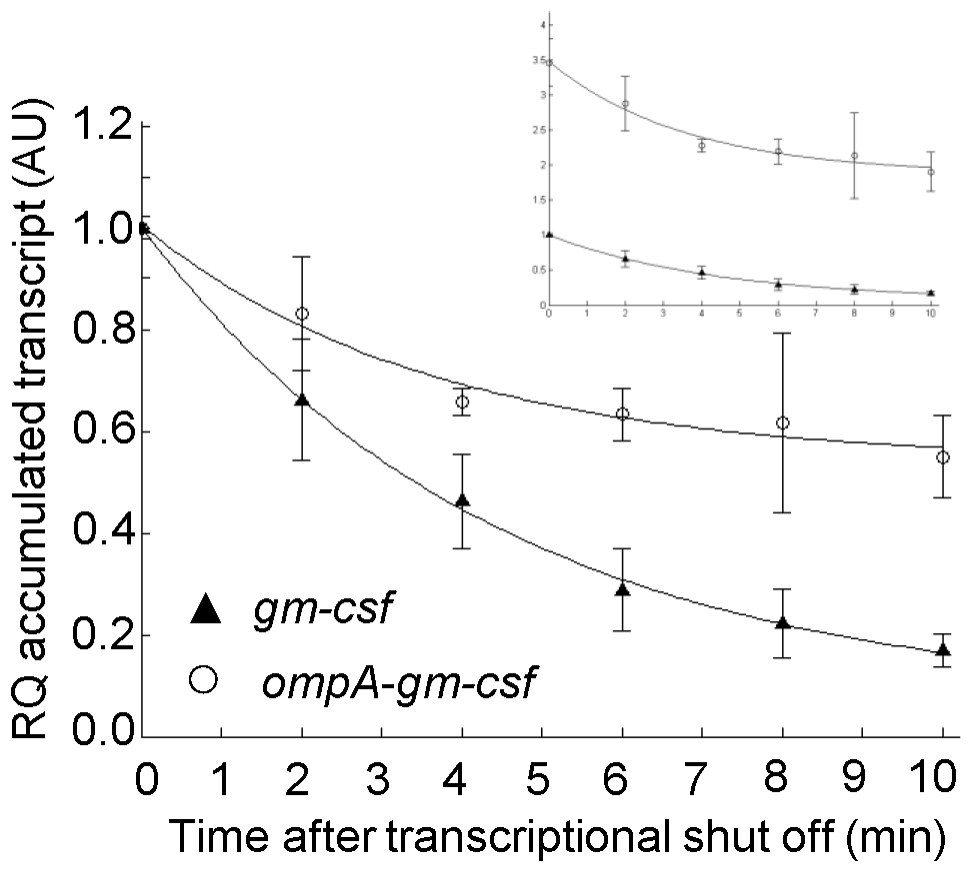
Kinetics of *gm-csf* and *ompA-gm-csf* transcript decay, expressed from ***Pm***. The larger plot shows decay kinetics with both *gm-csf* and *ompA-gm-csf* transcript levels at time point zero arbitrarily set to one. The smaller plot shown in the upper right corner represents all transcript data relative to the value of *gm-csf* at time point zero (arbitrarily set to one). Solid lines represent the best fit to the data calculated according to Eqn 4 (Material and Methods). Error bars show the deviation between two biological recurrences. RQ: relative quantification, AU: arbitrary units.

In a previous study [15], we showed that expression of the *gm-csf* gene, encoding human granulocyte-macrophage colony-stimulating factor (GM-CSF), is strongly stimulated at the transcript and protein levels, by fusing the *ompA* translocation signal sequence in frame with the 5′ end of the *gm-csf* coding sequence. This therefore represented a different test case for investigation of mRNA stability and its potential role in recombinant protein production. The decay of *gm-csf* transcript was monitored in parallel for DH5α (pGM29) and DH5α (pGM29ompA) strains. The results showed that the addition of *ompA* 5′ signal sequence leads to a significant increase in the *gm-csf* mRNA stability (Figure 4), estimated to be a 4-fold decrease in the relative decay rate; 0.31 for *gm-csf* (95% CI = 0.30–0.33) and 0.08 for *ompA-gm-csf* (95% CI: 0.08–0.09).

In addition, we did a similar test on the *ifn-α2b_S_* gene (encoding interferon *α*2b cytokine) fused to another translocation signal sequence, *pelB*. The basis for choosing this example was also the previous study [15], in which it was found that expression at the protein level was strongly improved by incorporating the signal sequence. Here we found that transcript amounts also increased up to 3.5-fold for the *pelB-ifn-α2b_S_* transcript (20 minutes after the induction), compared to the corresponding *ifn-α2b_S_* transcript lacking the *pelB* signal (data not shown). Based on monitoring the mRNA decay (data not shown), the relative decay rates were calculated to be 0.21 for *ifn-α2b_S_* (95% CI = 0.19–0.22) and 0.11 for *pelB-ifn-α2b_S_* (95% CI = 0.10–0.12). In summary, these results showed that two different 5′ signal sequences, acting on different genes, in both cases lead to enhanced mRNA stability. The corresponding transcripts are therefore inherently more stable than their counterparts lacking the signal sequences, or alternatively the signal leads to stimulation of translation, which in turn protects the transcript from degradation.

### The Inducer Wash-out Method can also be Applied to Study Decay Kinetics of mRNAs Produced from an IPTG Inducible Expression System

To broaden the range of possible applications of the inducer wash-out method we have included also an investigation of the commonly used IPTG-inducible LacI*/P_tac_* expression cassette, using the *gm-csf* gene with and without *ompA* 5′ fusion (constructs pFLAG-GM and pFLAG-OGM, respectively). To reduce background expression from the *P_tac_* promoter in the absence of inducer to a minimum we used a particular NEB Express Iq strain as host (Table 1). It was confirmed by GC-MS analyses that IPTG was efficiently removed by the filtration step. We further determined (by using SDS PAGE/Western blot analysis) that also in the pFLAG expression vector context the 5′ fusion is necessary for detectable protein production (data not shown).

**Figure 5:**
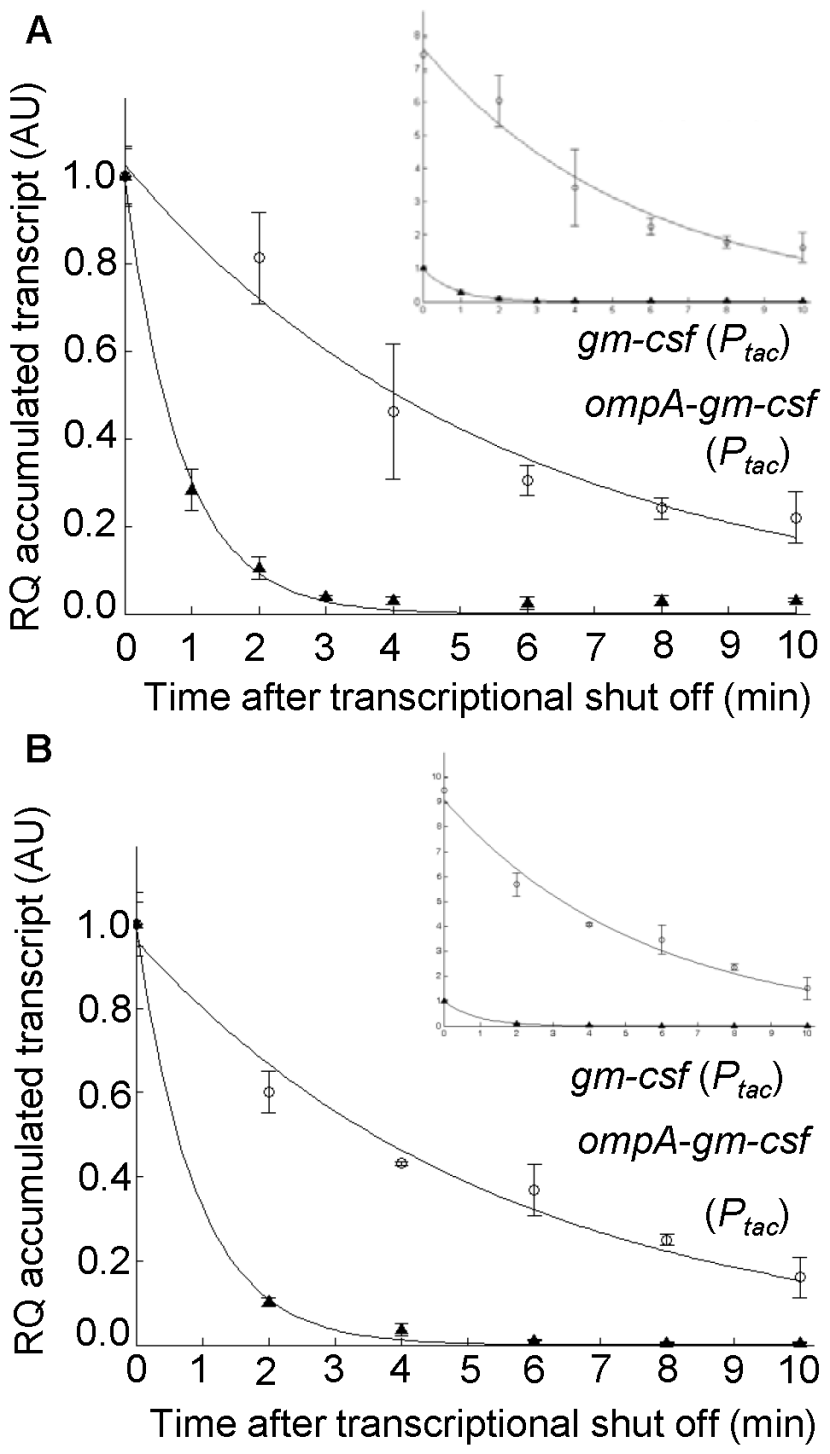
Decay kinetics for *gm-csf* and *ompA-gm-csf* transcripts expressed from *P_tac_*. **A.** *gm-csf* and *ompA*-*gm-csf* genes was achieved by the inducer wash-out. **B.** Rifampicin was used for transcription inhibition. For both A and B: the larger plot shows *gm-csf* and *ompA*-*gm-csf* transcript amounts at time point zero arbitrary set to one. In the upper right corner the decay curves are represented with all values relative to *gm-csf* transcript level at time point zero (arbitrarily set to one). Solid lines represent the best fit to the data calculated according to Eqn 4 (Material and Methods). Error bars show the deviation between at least three technical recurrences. RQ: relative quantification, AU: arbitrary units.

The NEB Express Iq (pFLAG-GM) and NEB Express Iq (pFLAG-OGM) strains were next used for determination of the corresponding mRNAs accumulation and decay over time. Comparison of the *ompA-gm-csf* and *gm-csf* transcript amounts revealed similar finding as when using the XylS/*Pm* system [15]; the *ompA-gm-csf* transcript amount produced from the *P_tac_* promoter was several-fold increased (about 6-fold 20 minutes after induction) compared to the *gm-csf* transcript amount (data not shown). Consistent with the initial observation for the XylS*/Pm* system, the presence of *ompA* 5′ fusion led to a significant increase in the *gm-csf* mRNA stability (Figure 5A); the relative decay rates were determined to be 1.27 for *gm-csf* (CI 95% = 1.19–1.35) and 0.43 for *ompA-gm-csf* (CI 95% = 0.38–0.46). The predicted relative decay rates differ by about factor 4 from the values determined for the same transcripts produced from the XylS/*Pm* system (see previous result chapter). However, in both the XylS/*Pm* and the LacI*/Ptac* systems, the *ompA* signal sequence was found to contribute to a 3–4 -fold decrease in the *gm-csf* relative decay rate. The differences in relative decay rates can be hypothetically caused by the 5′ UTR sequence following either the *P_tac_* or *Pm* promoter, and/or differences in mRNA degradation machinery of the *E. coli* strains used (NEB Express Iq versus DH5α).

These results therefore clearly demonstrated that the wash-out method can be used also for IPTG-inducible systems, which are probably the most heavily used of all inducible expression systems. We conclude that the methodology reported here is useful for analysis of recombinant mRNA stability in *E. coli* and presumably also in many other microorganisms in which similar inducible systems can be used, and both the XylS/*Pm* and the IPTG-inducible systems are themselves examples of expression cassettes that can be applied in other hosts [42]–[45].

### Selective Inhibition of Transcription by the Inducer Wash-out can be Used to Replace the Approach Based on Global Inhibition of Transcription by Rifampicin

A comparison of the inducer wash out strategy with the conventional method based on rifampicin was performed next. Promoters that are transcribed by the RNA polymerase core enzyme (E) associated with σ^S^ subunit are known to be more resistant to rifampicin than Eσ^70^ promoters [46]. Preliminary results with the *Pm* promoter, which is transcribed by Eσ^32^ in the exponential phase and by the Eσ^s^ in the stationary phase [47], showed that transcript production was not blocked by adding the commonly used concentration (200 mg/L) of rifampicin to the cell cultures (data not shown). Therefore, we chose to perform the rifampicin experiment with the *P_tac_* promoter (an Eσ^70^ promoter) to minimize the amount of rifampicin necessary to inhibit transcription and thereby minimize the stress level of the cells.

The same *E. coli* NEB Express Iq strains (pFLAG-GM and pFLAG-OGM) that were used to demonstrate the utilization of the LacI*/P_tac_* system were employed and the culture growth and induction conditions were kept the same as in the inducer wash-out experiment. The results showed that global inhibition of transcription in the cells leads to principally the same outcome as selective stopping of transcription of the *ompA-gm-csf* and *gm-csf* genes by the inducer wash-out (Figure 5B) under the conditions tested. The relative rate of *ompA-gm-csf* mRNA decay was about 2-fold lower than in the wash-out experiment (value of 0.18 with CI 95% = 0.16–0.21) while the decay rate of *gm-csf* mRNA differed only slightly (value of 1.11 with CI 95% = 0.97–1.25). We could speculate that most likely our simple decay model performs better in the case of the wash-out, which inhibits only transcription of the targeted mRNA. The cell-wide transcription shut-off brought about by rifampicin could introduce unknown effects that a simple mRNA decay model does not accommodate. In conclusion, although rifampicin based methods have proven invaluable for studying global mRNA decay patters [48], [49], the wash out method should allow for detail analysis of decay patterns of individual mRNAs. This can be found especially useful in gene engineering studies and in studying the effects of specific mutations, for example in the UTR or coding sequence regions.

### Analysis of the Bacteriophage T7 RNA Polymerase/promoter System

**Figure 6:**
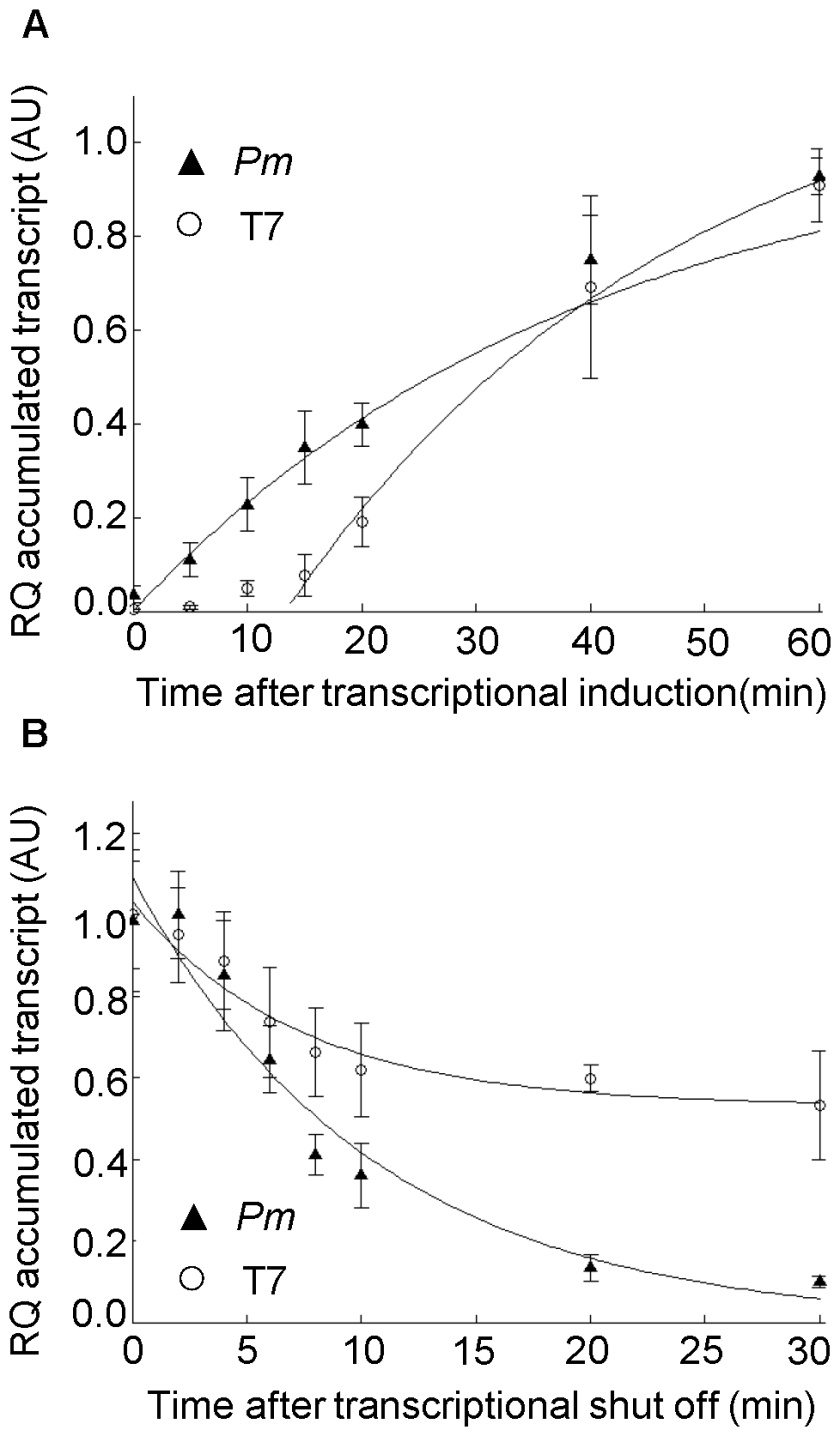
Accumulation and decay of *IL1RN_S_* transcripts. The respective genes were expressed from either the T7 or the *Pm* promoter. **A.** For both T7 and *Pm* promoter generated transcripts the amounts are given relative to the value at time point 60 minutes (arbitrarily set to one). The amounts of *IL1RN_S_* transcript generated through the T7 system was increased about 10-fold compared to those of the *Pm* system (after 60 minutes). Solid lines represent the best fit to the data, calculated according to Eqn 2 (Material and Methods). In case of the T7 system, only time points 15, 20, 40 and 60 minutes were included for the generation of the transcript accumulation curve. **B.** Parallel determination of decay for *Pm* and T7 generated transcripts. For both systems all transcript amounts are presented relative to *IL1RN_S_* transcript level at time zero (arbitrarily set to one). Error bars show the deviation between three biological recurrences. Solid lines represent the best fit to the data calculated according to Eqn 4 (Material and Methods). RQ: relative quantification, AU: arbitrary units.

Most of the results of the studies reported above were unpredictable beforehand, and to further verify the wash-out methodology we also decided to carry out an analysis with a more predictable outcome. This involved an investigation of the widely used and IPTG-inducible bacteriophage T7 RNA polymerase/promoter expression system (herein referred to as the T7 system). The genetic arrangement of this system implies that induction by IPTG first leads to synthesis of the T7 polymerase and in the second step to transcription of the target gene. In the vector design used here IPTG induction plays a dual role, as the T7 polymerase specific promoter (expressing the target gene) is also regulated by the LacI repressor. This control at two levels keeps expression of the target gene at a very low level in the absence of IPTG. Thus, in this case one would expect detectable target gene transcript only after a short delay representing the time needed to produce sufficient amounts of the polymerase. The effect of the sequential gene activation in the T7 system on recombinant transcript accumulation kinetics could be directly observed and compared to that of XylS/*Pm* by using *E. coli* ER2566 strains harboring analogous expression vectors pSB-E2r and pSB-M2r (Table 1). Both vectors express the synthetic codon-optimized gene for human interleukin 1 receptor antagonist (*IL1RN_S_)*, either from the T7 promoter (pSB-E2r) or from *Pm* (pSB-M2r). As also previously observed by [14], Figure 6a shows that expression from *Pm* starts immediately after inducer addition, while for the T7 polymerase dependent construct there is a clear lag in the transcript accumulation during the first minutes after induction. This presumably reflects the time needed to synthesize sufficient amounts of the T7 polymerase, and we therefore conclude that the qRT-PCR method is capable of visualizing this effect.

By similar reasoning we predicted a rather slow decrease in transcript amounts after inducer wash-out, since the T7 polymerase would likely be reasonably stable and would therefore allow continued transcription after IPTG removal. However, the situation is complex since pre-existing T7 polymerase action will be inhibited by the reactivation of LacI. Therefore the apparent decay reflects a mixture of actual decay and some level of new transcript synthesis. Indeed, the apparent decay of *IL1RN_S_* transcripts after removal of the IPTG inducer was found to be much slower in the T7 system compared to that of the *Pm* system. The remaining level of transcripts stayed over 50% of the start level even after 30 minutes of incubation in the absence of IPTG (Figure 6b).

## Discussion

This study presented a qRT-PCR-based approach that can be used to monitor recombinant mRNA decay in expression systems induced by low-molecular weight molecules whose transport into and out of cells is by diffusion. This rather simple technique only minimally affects the treated cells, in contrast to most other methods for accessing mRNA stability, which involve global inhibition of the bacterial RNA polymerase by the antibiotic rifampicin [50]. The latter approach may lead to possibly unknown negative side effects and in addition rifampicin-insensitive promoters have been described [48].

The analysis of *bla* associated 5′-UTRs directly showed that the stimulation of the transcript level by the LV-2 and LII-11 variants is based on enhanced rate of transcription and not more stable mRNA (Figure 1). Also as documented by the LII-11 variant, more efficient translation does not always result in better protection against transcript degradation, as has been reported for other genes [37], [51]. However, in accordance with those reports, we showed here that a translocation signal sequence fused in frame to the 5′ end of a human gene can stimulate its mRNA stability (Figure 4). These unpredictable differences presumably are a consequence of the complexity of the relations between transcription, mRNA decay and translation. Adding a 5′ signal sequence may have an impact on mRNA folding and then may or may not affect RNase E-mediated mRNA decay. Alternatively, the observed increase in the transcript amount and prolonged mRNA half-lives in the case of 5′ fusions may indicate a possible protection of the transcripts by translating ribosomes due to more efficient translation initiation. Further studies, for example by the use of a novel technique for quantitative analysis of the ribosomal density on translated mRNAs [52], could potentially be used to gain more detailed insight, but generally we believe that it is very complicated to unravel the underlying mechanisms by currently available techniques.

Similarly to the *bla* associated 5′-UTR variants, introduction of synonymous mutations in the *bla* 5′ coding sequence led to an increase in the accumulated transcript level without evidently affecting mRNA stability (Figure 3). The reasons for this may be similar to those underlying the effect of the LV-2 UTR mutations [14], but it is interesting that the coding sequence of a gene can have such an impact on transcription itself. Even though the 5′ coding region has been linked multiple times to the control of translation through its mRNA secondary structures and codon usage [53], [54] much less is known about how it can affect transcription. It has been shown that the adjacent 5′-UTR DNA region can influence the transcriptional process through sequences resembling the −10 promoter element (TATAAT) that are able to induce σ^70^-dependent transcriptional pausing [55], [56]. In case of the tryptophanase operon (*tna*), transcriptional pause sites have been described in the 220-nucleotides long spacer region separating the coding regions of TnaC leader peptide and TnaA tryptophanase, and implicated in coupling of translation with transcription [57]. One possibility is therefore that the synonymous mutations within the *bla* coding region affect pause sites in the wild type *bla* DNA sequence in a way that is stimulatory for transcription.

To substantiate the general relevance of the new methodology for assessment of mRNA stability, we expanded its use also to an IPTG-inducible promoter. The decay of mRNAs (*ompA-gm-csf* and *gm-csf*, Figure 5) generated from the *P_tac_* promoter confirmed that the methodology was equally useful for this IPTG-inducible system as for the *m*-toluate-inducible XylS/*Pm* system. One potential limitation of this method might be that it requires the use of an inducible promoter with low background expression level. Any background production of mRNA under study would likely lead to a mixture of actual decay and some level of new transcript synthesis, causing an error in the calculation of the relative decay rate.

The inducer wash-out method was tested with plasmids having both low (e.g. pIB11) and moderately high copy number (e.g. pGM29). Given that the qRT-PCR is generally regarded as a highly specific and sensitive technique we believe that the method can without difficulty address different expression levels. Moreover, when used together with determination of the transcript levels and the respective protein amount the inducer wash-out method should be useful for elucidating different factors that might be affecting overall expression level of a recombinant gene. The concept described here can most likely be used to measure mRNA decay for any gene in basically any cell type (even some eukaryotes) that is heavily used in molecular biology research. The reason is that systems based on diffusible inducers are almost universally available. Whatever the gene of interest is it can simply be PCR-amplified with its own UTR and then inserted downstream of an inducible promoter. This will likely give the same transcript as from the natural environment in which the gene is localized.
